# Supplementary material for: Simultaneous stabilization of actin cytoskeleton in multiple nephron-specific cells protects the kidney from diverse injury
Source: Nat Commun. 2022 May 3;13:2422. doi: 10.1038/s41467-022-30101-4 (PMC9065033; doi:10.1038/s41467-022-30101-4)
Supplement: Supplementary file 1 — Supplementary Information [file 41467_2022_30101_MOESM1_ESM.pdf]

Simultaneous stabilization of actin cytoskeleton in multiple nephron-specific cells protects the kidney from diverse types of injury

Kamalika Mukherjee[1#], Changkyu Gu[1#], Agnieszka Collins[1#], Marcel Mettlen[2], Beata Samelko[3], Mehmet M. Altintas[3], Yashwanth R. Sudhini[3], Xuexiang Wang[3], Richard Bouley[1], Dennis Brown[1], Bradley P. Pedro[1], Susan L. Bane[4], Vineet Gupta[3], Paul T. Brinkkoetter[5,6], Henning Hagmann[5,6], Jochen Reiser[3*] and Sanja Sever[1*]

[1]Department of Medicine, Harvard Medical School and Division of Nephrology, Massachusetts General Hospital, Boston, MA

[2]Department of Cell Biology, University of Texas Southwestern Medical Center, Dallas, TX

[3]Department of Medicine, Rush University Medical Center, Chicago, IL

[4] Department of Chemistry, Binghamton University, State University of New York, Binghamton, NY

[5]Department of Internal Medicine-Center for Molecular Medicine Cologne, University of Cologne and Faculty of Medicine-University Hospital Cologne, Cologne, Germany

[6]Cologne Cluster of Excellence on Cellular Stress Responses in Ageing-Associated Diseases (CECAD) and Systems Biology of Ageing Cologne (Sybacol), Cologne, Germany

*Corresponding Authors:

E-mail: ssever@mgh.harvard.edu

E-mail: Jochen_reiser@rush.edu

[#] These authors contributed equally

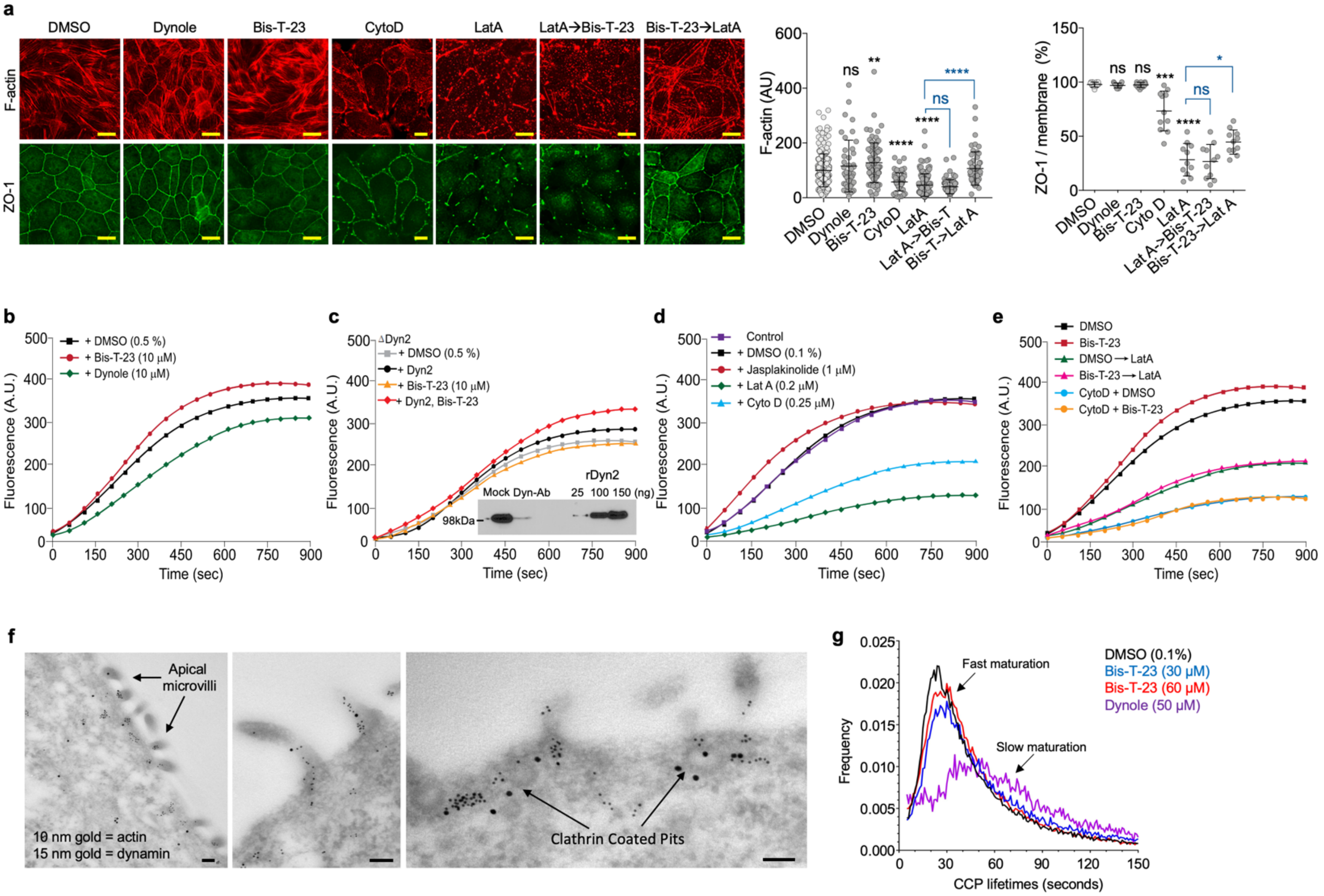

Figure S1. Dynamin agonist Bis-T-23 affects the actin cytoskeleton independently from endocytosis. **a** MDCK cells were treated with DMSO (0.1%), Dynole (10 μM), CytoD (0.25 μM), Bis-T-23 (30 μM), or LatA (0.2 μM) for 20-30 min. When the interplay between Bis-T-23 and LatA was examined, cells were treated with identical concentrations of Bis-T-23 or LatA for 10 min prior to the addition of LatA or Bis-T-23 for another 20 min or 10 min, respectively. F-actin was stained with phalloidin (red) and ZO-1, representing tight junctions, was stained with an anti-ZO-1 antibody (green). Scale bar, 20 μm. As some treatments generate actin patches along the cell membrane, F-actin intensity (44-211 cells per treatment) shown in the graph (AU, arbitrary units) was measured within the central region as described in *Methods*. ZO-1 (11 cells per treatment) is presented as a percentage of the total membrane length (%). Error bars, mean ± S.D. (**P*<0.05, ***P*< 0.01, ****P*< 0.001, *****P*<0.0001, one-way ANOVA with Tukey's multiple comparisons test). ns, not significant. **b-e** Solution-based actin polymerization assays using cell extracts isolated from MDCK cells. Shown are representatives of three independent experiments. **b** Indicated concentrations of DMSO, Bis-T-23, or Dynole were added to protein extracts for 10 min prior to the initiation of actin polymerization. **c** Cell extracts were depleted of endogenous Dyn2 using a polyclonal anti-dynamin 2 antibody (ΔDyn2). When indicated, the endogenous amount of recombinant Dyn2 (20 nM) was added to extracts. Efficacy of immunodepletion was assessed by Western blot analysis (inset). Equal volumes of cell extracts incubated with either non-specific IgG or polyclonal anti-dynamin 2 antibodies were probed using a monoclonal anti-dynamin antibody. Increasing concentrations of recombinant Dyn2 (rDyn2) were used as a standard. **d** Indicated concentrations of DMSO, Jasplakinolide, LatA or CytoD were added to cell extracts for 10 min prior to initiation of actin polymerization. **e** When indicated, cell extracts were treated with DMSO (0.5%) or Bis-T-23 (10 μM) for 30 min. Alternatively, cell extracts were treated with identical concentrations of DMSO or Bis-T-23 for 10 min prior to the addition of LatA (0.2 μM) for an additional 20 min. In addition, extracts were simultaneously treated with CytoD (0.25 μM) and DMSO (0.5%) or CytoD (0.25 μM) and Bis-T-23 (10 μM) for 30 min. **f** Immunogold electron microscopy of the apical membrane of MDCK cells identifying co-localization of dynamin and actin using monoclonal anti-dynamin (15 nm gold) and polyclonal anti-actin (10 nm gold) antibodies. Dynamin and actin co-localized within the microvilli, at the actomyosin cortex just beneath the plasma membrane, and at the clathrin-coated pits, identified by their distinct shape and size. Scale bars, 100 nm. **g** MDCK cells

stably expressing eGFP-tagged clathrin light chain were treated with indicated concentrations of DMSO, Bis-T-23 or Dynole for 30 min prior to data collection. Distribution of clathrin-coated pits (CCPs) lifetimes at the plasma membrane were determined using TIRFM and automated tracking of CCPs. Data were generated from over 100 cells, and a range of 0.5-1.0$\times10^6$ CCPs were analyzed per treatment. Note that the distribution of the CCPs lifetimes (speed by which CCPs are maturing at the plasma membrane) was similar in cells treated with DMSO and Bis-T-23, with a majority of the CCPs in the 30 second range: the time from initiation of the assembly of the clathrin coat to the moment that a mature vesicle is released from the membrane. Dynole treatment significantly decreased the number of CCPs with shorter lifetimes and increased the number of CCPs with longer lifetimes, consistent with the inhibitory effect of Dynole on dynamin's role in endocytosis.

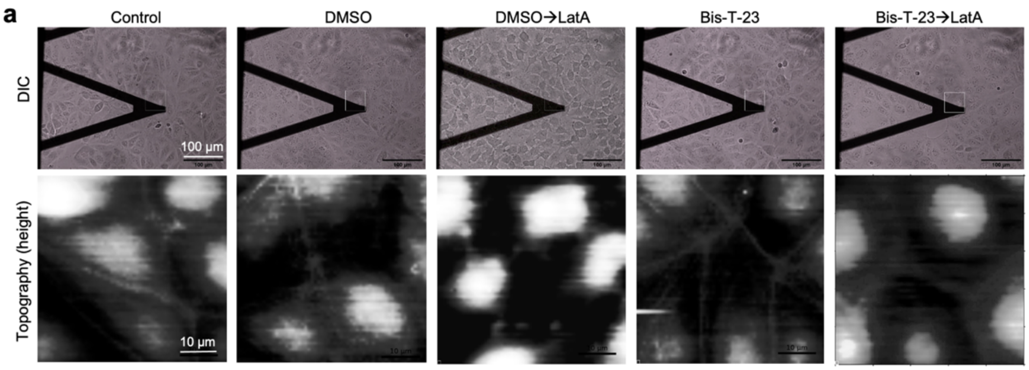

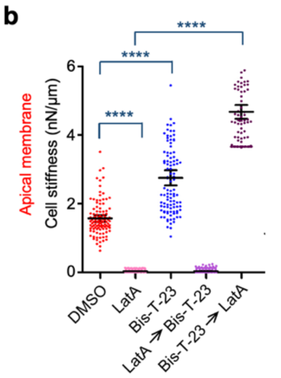

c

Treatment	Cell stiffness (nN/µm)
DMSO	1.57±0.50
LatA	0.03±0.03*
Bis-T-23	2.75±1.14
LatA→Bis-T-23	0.03±0.05 (P>0.05)*
Bis-T-23→LatA	4.68±1.15 (P<0.0001)*

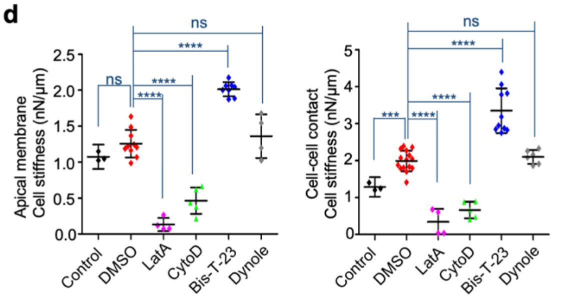

e

Treatment	Cell stiffness (nN/µm)	
	Apical	Cell-cell contact
Control	1.07±0.07	1.29±0.11
DMSO	1.26±0.19	1.99±0.28
LatA	0.13±0.09 (P<0.0001)	0.34±0.35 (P<0.0001)
CytoD	0.46±0.18 (P<0.0001)	0.67±0.23 (P<0.0001)
Bis-T-23	2.01±0.10 (P<0.0001)	3.35±0.61 (P<0.0001)
Dynole	1.36±0.30 (P>0.05)	2.10±0.19 (P>0.05)

Figure S2. Dynamin agonist alters cell stiffness in an actin-dependent manner.
a Representative images of MDCK cells grown to confluency that were used to acquire Young's Modulus maps by AFM using the NanoWizard IV system and their respective topography maps (see also Fig. 1c). MDCK cells were treated with DMSO (0.1%) or Bis-T-23 (30 µM) for 30 min prior to data acquisition. When indicated, cells were treated with Bis-T-23 (30 µM) or DMSO (0.1%) for 10 min prior to the addition of LatA (0.2 µM) for 20 min. **b** Scattered graphs showing cell stiffness in MDCK cells treated as described in (**a**) and also in cells that were treated with identical concentrations of LatA for 10 min followed by Bis-T-23 for 10 min. Data were acquired with the BioScope II system. Each symbol represents a single force curve acquired at the apical membrane. Values were generated by 3 independent experiments in 4-7 individual cells. Error bars, mean ± S.E.M. (*****P*<0.0001, unpaired two-tailed *t*-test). **c** Table presenting mean ± SD for data shown in (**b**). Statistical significance was determined using an unpaired two-tailed *t*-test for comparison between LatA-only treated cells and those sequentially treated with LatA and Bis-T-23. **d** MDCK cells were treated with DMSO (0.1%), LatA (0.2 µM), CytoD (0.25 µM), Bis-T-23 (30 µM) or Dynole (10 µM) for 30 min prior to data acquisition. AFM was performed using BioScope II. Each symbol represents the average stiffness determined by the value of force indentation curves at a single cell-cell contact or at the apical region of the single cell. Values were generated by 3 independent experiments. Error bars, mean ± S.E.M. (*****P*<0.0001, unpaired two-tailed *t*-test). ns, not significant. Note, cell-cell contact stiffness observed in cells treated with DMSO or Bis-T-23 is slightly different in Fig 1d and Fig S2d. These differences may be due to variations in experimental parameters, such as cell density and/or the duration of cells in culture. Importantly, identical trends for Bis-T-23-mediated preservation of LatA injury are seen at the apical membrane and at cell-cell contact in both Figures regardless of the experimental technique used. **e** Table presenting mean ± SD for data shown in (**d**). Statistical significance between DMSO treated cells and LatA, CytoD, Bis-T-23 or Dynole treated cells was determined using an unpaired two-tailed *t*-test.

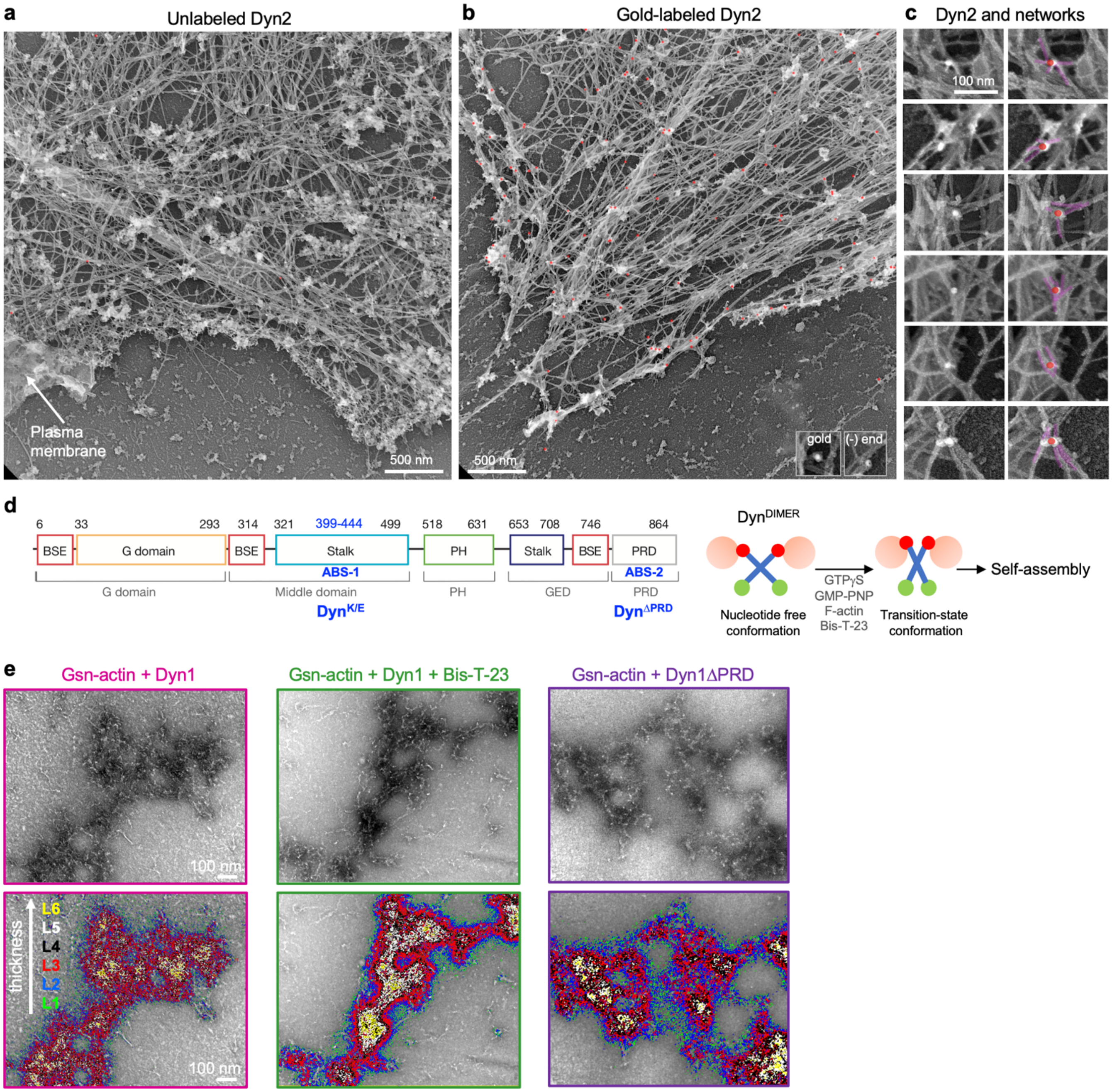

Figure S3. Dynamin localizes on the actin network in MDCK cells.
a-**c** Immunogold platinum replica electron micrographs focusing on actomyosin cortex in MDCK cells. Cellular localization of endogenous Dyn2 was determined by monoclonal anti-Dyn2 antibody and gold-conjugated secondary antibody (18 nm, white particles). In a control experiment, cells were labeled only with anti-dynamin antibodies (**a**). To better visualize the gold particles within tightly packed actin networks, white densities associated with gold particles were pseudo-colored in red in (**b**) and (**c**). Actin filaments associated with gold particles were pseudo-colored pink in (**c**). Notice that dynamin associates with multiple actin filaments, consistent with its newly identified ability to crosslink F-actin into networks using a reconstituted system. **d** Upper panels show representative micrographs of actin arrays assembled from Gsn-actin and Dyn1 or Dyn1ΔPRD in the presence or absence of Bis-T-23 (0.4 µM). The lower panel shows contour plots depicting density as varying depths within a network. Distinct levels of thickness are color-coded as indicated in the figure. Contour plots were produced based on the original EM images shown in the upper panel. Representative images of two independent experiments are shown here.

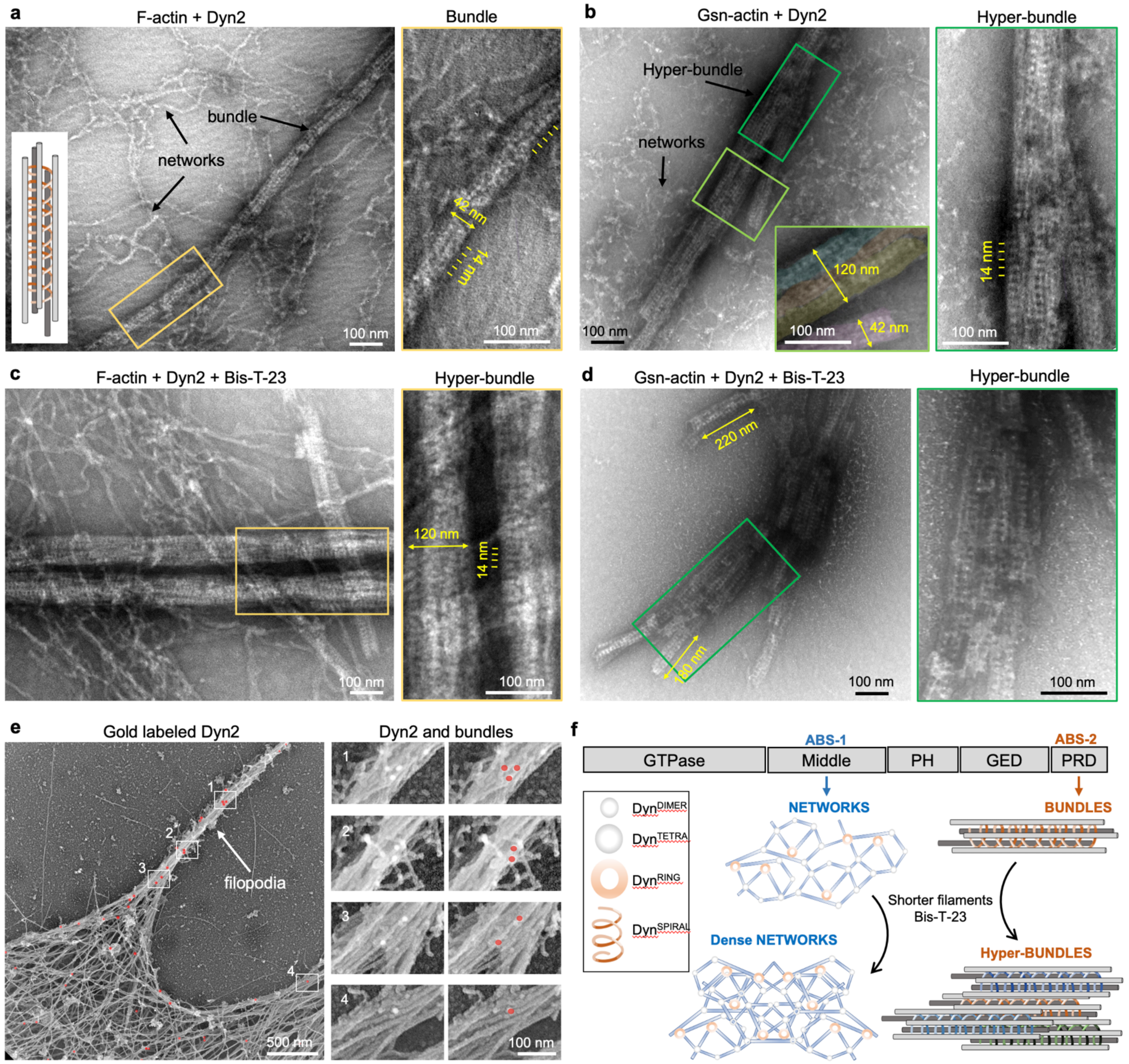

Figure S4. Dynamin crosslinks F-actin into bundles and hyper-bundles.

a, b Examples of negatively stained tight actin bundles formed by incubating Dyn2 with either long F-actin (**a**) or short Gsn-actin (**b**). A higher magnification image of boxed regions shows a strictly regular arrangement of tightly packed actin bundles. The average width of the single bundle was ~43 nm with a transverse periodicity of ~14 nm. The transverse pattern, associated with dynamin helices, occurred perpendicular to the bundle axis. The enclosed model in (**a**) depicts the mode of bundle formation by dynamin rings/helices. In addition, single bundles within the hyper-bundle are pseudo-colored in different colors in (**b**). The average width of a single hyper-bundle was ~120 nm. **c, d** Electron micrographs of actin bundles generated by Dyn2 and long F-actin (**c**) or short Gsn-actin (**d**) in the presence of Bis-T-23 (0.4 μM). The bundles' width and characteristic transverse repeats are indicated in the pictures. Data show that Bis-T-23 increased dynamin's ability to crosslink long and short filaments into hyper-bundles. This mechanism might underlie the formation of microvilli at the apical membrane of MDCK cells. **e** Immunogold platinum replica electron micrographs of MDCK cells focusing on actin bundles in filopodia. Cellular localization of endogenous Dyn2 was determined by monoclonal anti-Dyn2 antibody and gold-conjugated secondary antibody (18 nm, white particles were pseudo-colored in red). Higher magnification

images identify dynamin within parallel actin bundles, consistent with its appearance in the reconstituted system. Representative images of two independent experiments. **f** Schematic representation of dynamin domains and two distinct actin-binding sites: first associated within the Middle domain and second within the PRD. Dynamin uses PRD to assemble actin filaments into bundles, and the Middle domain for crosslinking filaments into networks. Diverse dynamin oligomerization states are represented by circles. Our study suggests that networks are formed by a combination of dynamin dimers, tetramers, and rings. In contrast, bundles are formed by dynamin helices. Dynamin's ability to crosslink actin filaments into networks and bundles is stimulated by Bis-T-23 and is more pronounced for the shorter filaments. Our study suggests that actin networks assembled by dynamin at the apical membrane are the major structural component that underlies polarity and cell stiffness in polarized epithelial cells. Dynamin's ability to assemble bundles does not seem to be essential for the establishment/maintenance of cell polarity but it is possible that it does contribute to the maintenance of overall cell integrity upon injury.

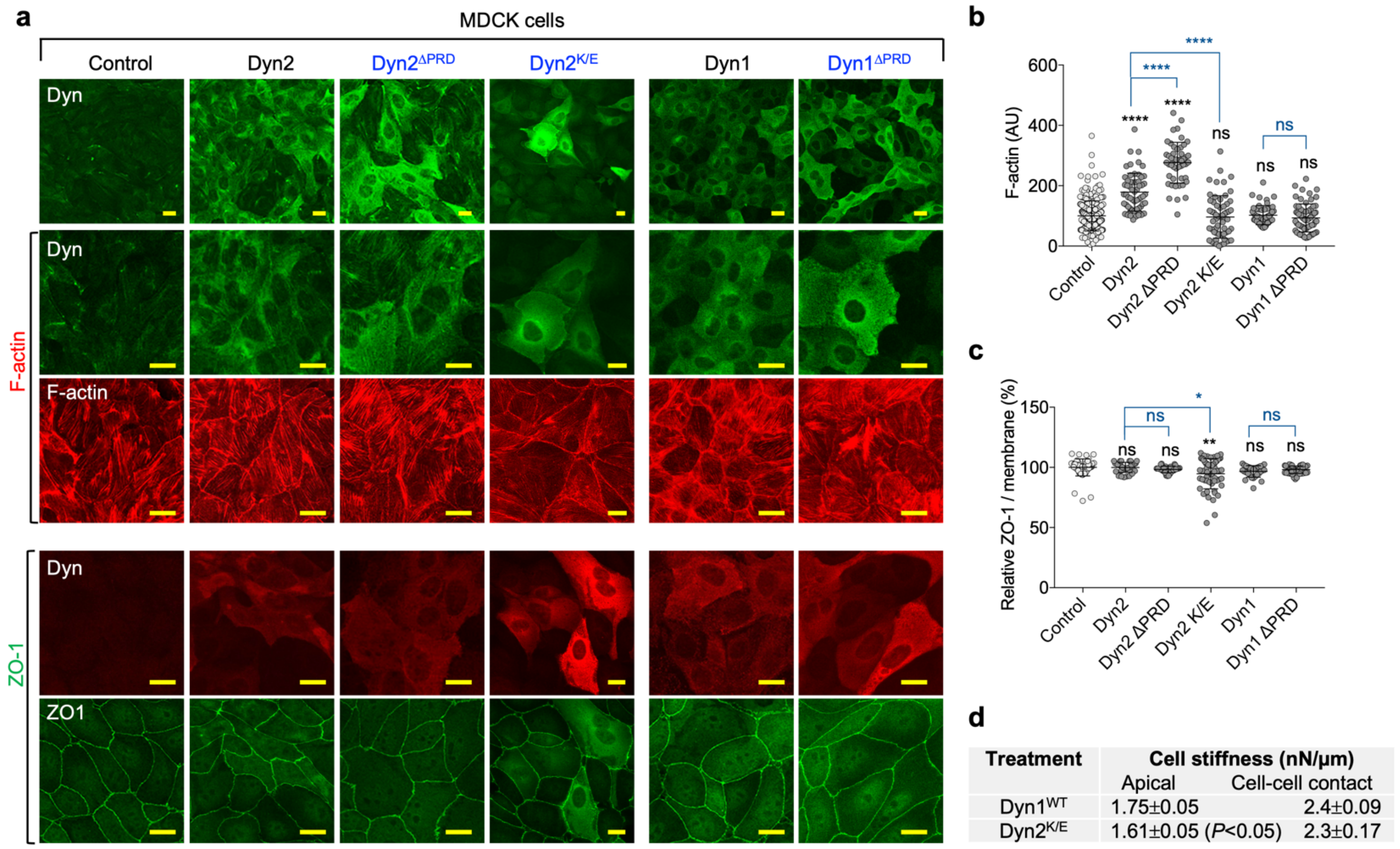

Treatment	Cell stiffness (nN/µm)	
	Apical	Cell-cell contact
Dyn1^{WT}	1.75±0.05	2.4±0.09
Dyn2$^{K/E}$	1.61±0.05 (*P*<0.05)	2.3±0.17

Figure S5. Dynamin's Middle domain but not PRD is involved in the maintenance of cell polarity.
a Representative image of MDCK cells expressing diverse dynamin proteins. The top panel shows the overexpression levels of dynamins as cells were stained with an anti-dynamin antibody (green). MDCK cells were co-stained with either an anti-dynamin antibody and phalloidin (F-actin) or an anti-dynamin antibody and anti-ZO-1 antibody. Scale bar, 20 µm. **b** Graph showing F-actin intensity (AU, arbitrary units) measured within the central region of a cell (50-289 cells). **c** Graph showing ZO-1 staining as a percentage of the total membrane length (%) relative to control cells (28-60 cells). Error bars mean ± S.D. (**P*<0.05, ***P*<0.01, ****P*<0.001, *****P*<0.0001, one-way ANOVA with Tukey's multiple comparisons test). ns, not significant. **d** Table showing mean ± S.E.M. for cell stiffness measured at cell-cell contact and at the apical membrane. Indicated dynamin proteins were over-expressed in MDCK cells and AFM was performed using BioScope II. Statistical significance between the two samples was calculated using an unpaired two-tailed *t*-test.

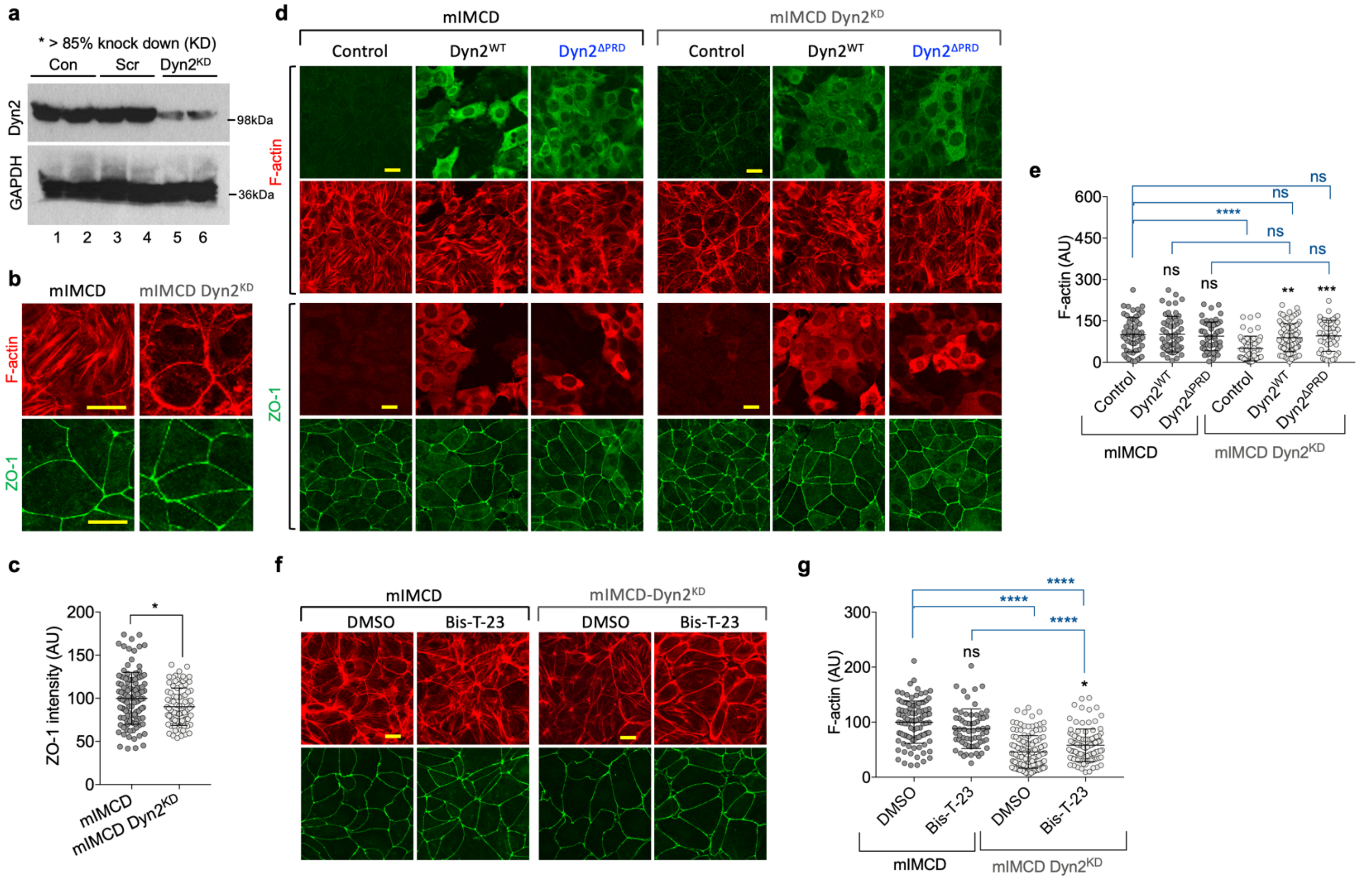

Figure S6. Dynamin influences cell polarity via its effect on the actin cytoskeleton.

a Representative Western blot showing the level of endogenous Dyn2 in mIMCD cell extracts in which Dyn2 was stably downregulated (mIMCD-Dyn2^{KD}). Cell extracts were generated from either wild-type mIMCD or cells that were infected with lentiviruses carrying shRNA for either mouse Dyn2 or scrambled sequence (see *Methods* section). Representative images of two independent experiments. **b** Representative images of confluent mIMCD and mIMCD-Dyn2^{KD} cells stained with phalloidin for F-actin (red) and anti-ZO-1 antibody (green). IMCD-Dyn2^{KD} cells exhibited loss of stress fibers within the central region of the cell and the formation of actin rings along the membrane. In addition, the ZO-1 staining pattern looked serrated, demonstrating that the loss of Dyn2 leads to major alterations in the overall organization of the actin cytoskeleton as well as cell polarity in mIMCD cells. Scale bar, 20 µm. **c** Scatter dot plot showing ZO-1 intensity (AU) within a fixed region of interest (ROI) at the cell membrane of mIMCD (cells=112) and mIMCD-Dyn2^{KD} cells (cells=87) shown in (**b**). Error bars, mean ± S.D. (*P<0.05, unpaired two-tailed *t*-test). **d** Representative images of mIMCD or mIMCD-Dyn2^{KD} cells co-stained with anti-dynamin antibody and phalloidin (F-actin), or anti-dynamin antibody and anti-ZO-1 antibody. Where indicated, cells adenovirally expressed Dyn2^{WT} or Dyn2$^{\Delta PRD}$. Note that expression of wild type dynamins or ΔPRD dynamins partially restored F-actin within the cell body and improved the smoothness of the ZO-1 staining pattern. Scale bar, 20 µm. **e** Graph showing F-actin intensity in an ROI within the central region of cells shown in (**d**). The data represent measurements of 49-85 cells. **f** Representative images of confluent mIMCD or mIMCD-Dyn2^{KD} cells stained with phalloidin (F-actin) and anti-ZO-1 antibody. Cells were treated with DMSO (0.1%) or Bis-T-23 (30 µM) for 30 min. Note that Bis-T-23 failed to induce a wild-type level of F-actin within the cell body of mIMCD-Dyn2^{KD}. Scale bar, 20 µm. **g** Graph showing F-actin intensity within the central region of the cell in cells shown in (**f**). The data represent measurements of 63-129 cells. Data in the figures **e** and **g** are plotted as mean ± S.D. (*P<0.05, ****P<0.0001, one-way ANOVA with Tukey's multiple comparisons test). ns, not significant.

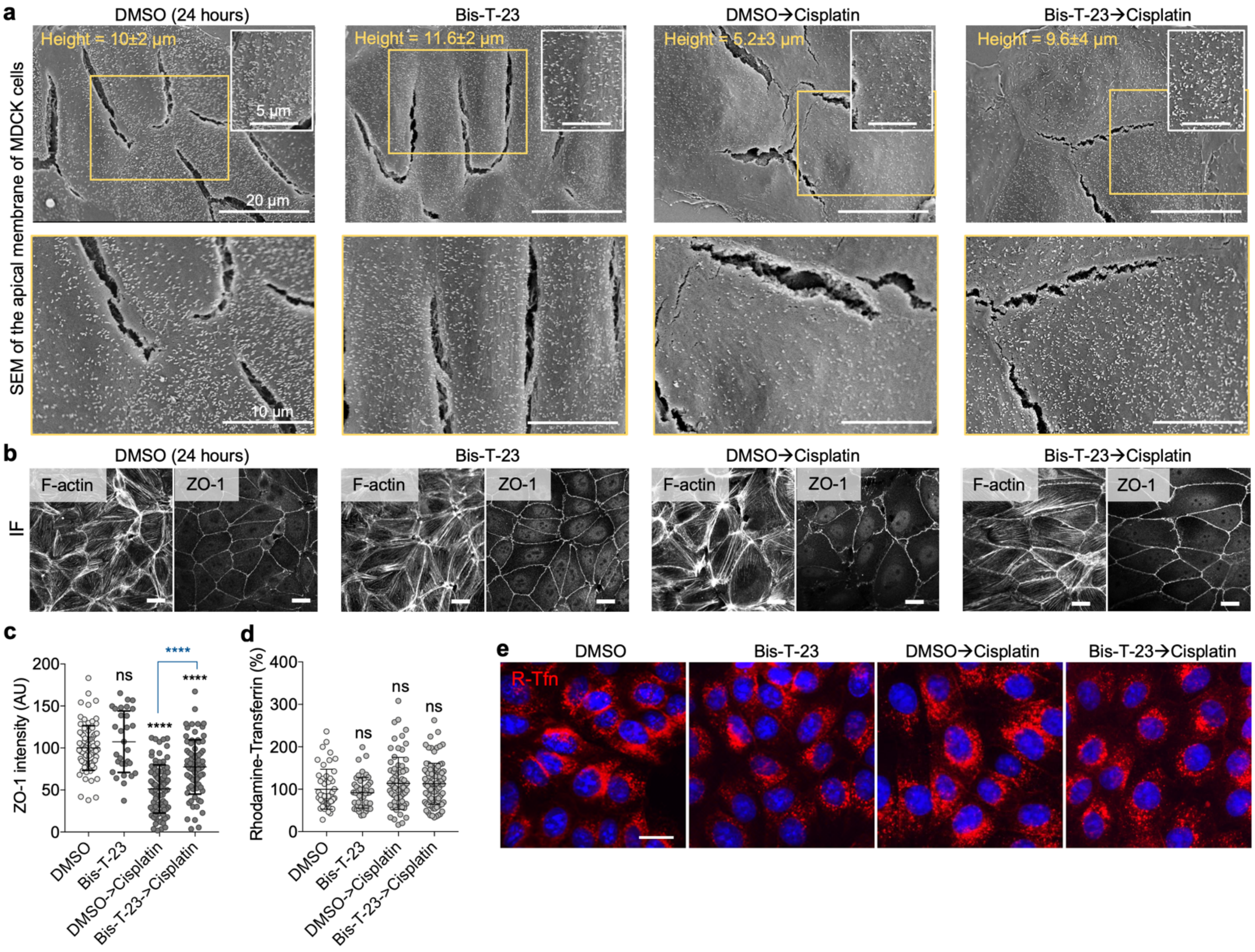

Figure S7. Bis-T-23 protects against cisplatin-induced loss of cell polarity in MDCK cells.
a Representative SEM image of MDCK cells treated with DMSO (0.1%) or Bis-T-23 (5 μM, 0.1%DMSO) for 24 hours. In the case of cisplatin treatment, cells were treated with identical concentrations of either DMSO or Bis-T-23 for 1 hour, after which cisplatin (35 μM) was added for an additional 23 hours prior to fixing the cells. Insets show the morphology of microvilli at higher magnification. Higher magnification orange-boxed images demonstrate microvilli arrangement, distribution, and density. Scale bars are indicated in the figure. **b** Representative images of MDCK cells stained with phalloidin (F-actin) and anti-ZO-1 antibody. Cells were treated as described in (**a**). Scale bar, 20 μm. **c** Scatter dot plots showing ZO-1 intensity (30-89 ROIs). Error bars, mean ± S.D. ($P<0.001$, one-way ANOVA with Tukey's multiple comparisons). ns, not significant. **d**, The level of endocytosed R-Tfn (AU, arbitrary units) relative to vehicle control (49-98 cells). Error bars, mean ± S.D., ns, not significant. MDCK cells were treated as described in (**a**) and were then allowed to endocytose R-Tfn in a fresh serum-free medium for 10 min. The nucleus was stained with Hoechst 33342 (blue) after fixing. **e** Representative images of internalized rhodamine-Transferrin (R-Tfn) in MDCK cells. Scale bar, 20 μm.

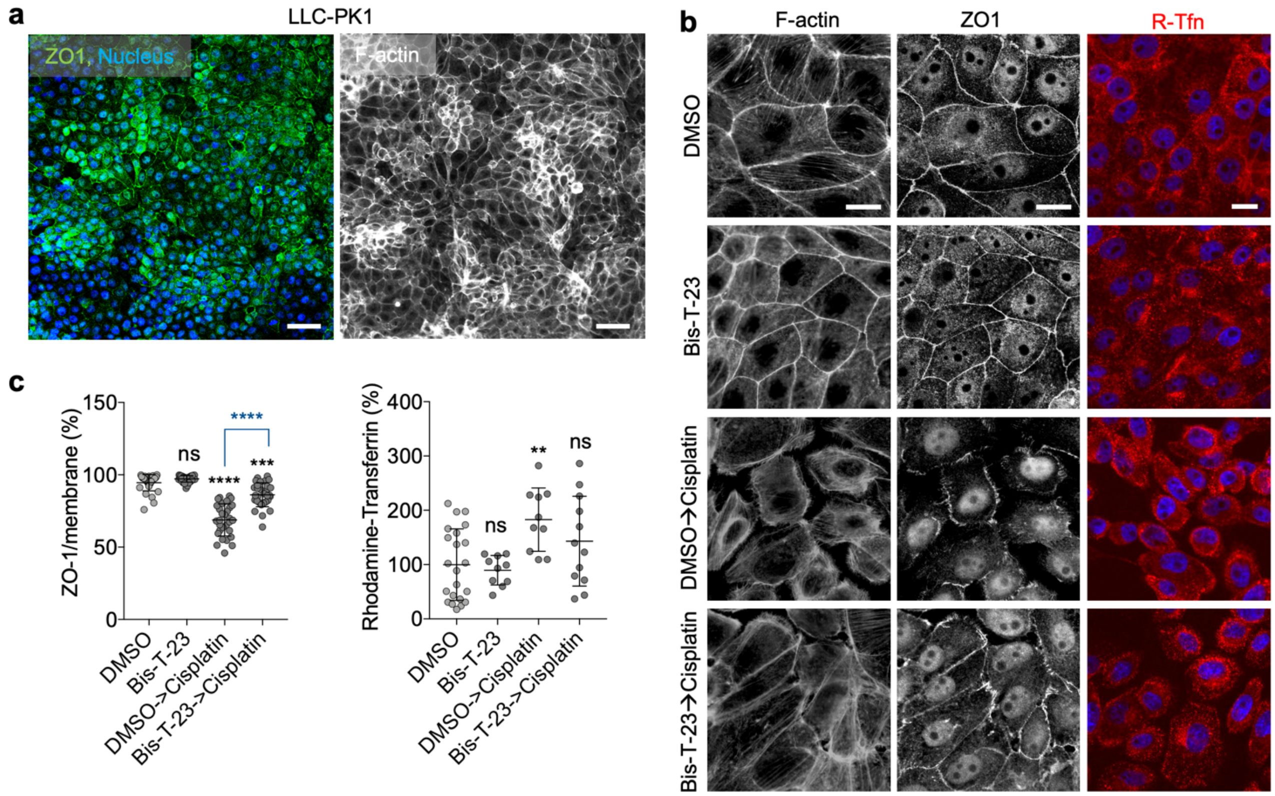

Figure S8. Bis-T-23 protects against cisplatin-induced loss of cell polarity in LLC-PK1 cells.
a Representative images of polarized LLC-PK1 cells stained with anti-ZO-1 antibody (green), Hoechst 33342 for nucleus (blue), and phalloidin (white) for F-actin. Scale bar, 50 μm. **b** Representative images of LLC-PK1 cells treated with DMSO (0.1%), cisplatin (10 μM), Bis-T-23 (10 μM, 0.1%DMSO), or a sequential combination of both. Cells were treated with DMSO or Bis-T-23 for 1 hour prior to the addition of cisplatin (10 μM) and incubated for an additional 23 hours. F-actin was stained with phalloidin, ZO-1 with an anti-ZO1 antibody. The level of endocytosis under identical conditions was determined by measuring the intensity of internalized R-Tfn (red). The nucleus was stained with Hoechst 33342 (blue). Scale bar, 20 μm. **c** Graph showing ZO-1 length as a percentage of total membrane length (%) (30-32 cells), and the level of endocytosed R-Tfn relative to the vehicle control (%) (10-22 cells). Error bars, mean ± S.D. (***$P<0.001$, ****$P<0.0001$, one-way ANOVA with Tukey's multiple comparisons test). ns, not significant.

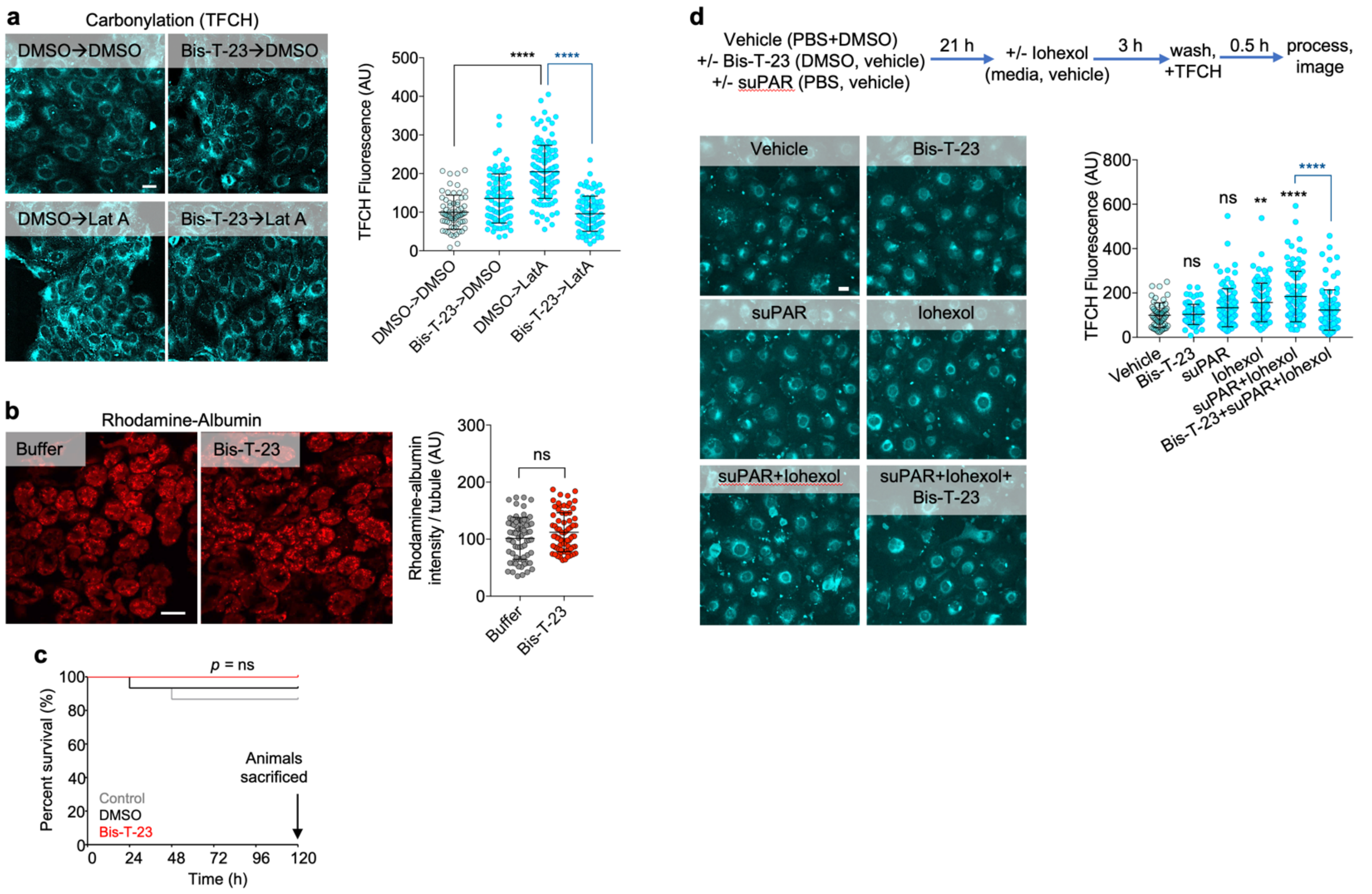

Figure S9. Bis-T-23 counteracts oxidative stress via its effect on actin network.
a Representative images and quantification (data points (n) = 67-122; each data point represents average intensity of 3-14 cells) showing the level of oxidative stress-induced carbonylation in MDCK cells using TFCH assay. The cells were treated with DMSO (0.1%) or Bis-T-23 (30 μM, 0.1% DMSO) for 20 min prior to the addition of DMSO (0.1%) or LatA (0.2 μM, 0.1% DMSO) for 10 min. Subsequently, TFCH (20 μM, 0.5% DMSO) was added for an additional 30 min. Scale bar, 20 μm. Graph depicting the level of oxidative stress-induced carbonylation under conditions described. Data are plotted as mean ± S.D. (*****P*<0.0001, one-way ANOVA with Tukey's multiple comparisons test). **b** Representative images, and quantification showing endocytosis of rhodamine albumin in rat kidney slices. Rat kidney slices were incubated with buffer or Bis-T-23 (30 μM) for 1.5 hours prior to adding rhodamine-albumin for 30 min. Graph showing internalized rhodamine-albumin per tubule (72 per condition) in buffer and Bis-T-23 treated kidney slices. Scale bar, 40 μm. Data are plotted as mean ± S.D. An unpaired two-tailed *t*-test was performed, ns, not significant. **c** Log-Rank (Mantel-cox) test was performed to analyze the survival curve of the two treatment groups that received Bis-T-23 and/or DMSO in addition to cisplatin and the control animals that received only cisplatin (see Fig. 4h). All animals were sacrificed at day 5. ns, not significant. **d** A schematic representation showing the sequential treatment of HK-2 cells with a combination of vehicle, Bis-T-23, suPAR and/or Iohexol. Representative images and quantification (data points (n) = 53-104; each point represents average intensity of 2-4 cells) showing the level of oxidative stress-induced carbonylation in HK-2 cells using TFCH assay. Scale bar, 20 μm. Data are plotted as mean ± S.D. (***P*<0.01, *****P*<0.0001, one-way ANOVA with Tukey's multiple comparisons test). ns, not significant.
